# Loneliness and Social Connectedness Among Rural Older Adults Since the COVID-19 Pandemic Onset

**DOI:** 10.1093/geroni/igab046.2206

**Published:** 2021-12-17

**Authors:** Gabriella Meltzer, Lindsay Kobayashi, Jessica Finlay, Carrie Henning-Smith

**Affiliations:** 1 Department of Social and Behavioral Sciences, New York University, New York, United States; 2 University of Michigan, Ann Arbor, Michigan, United States; 3 University of Minnesota School of Public Health, Minneapolis, Minnesota, United States

## Abstract

Rural areas have a higher proportion of older adults aging in place. Rural areas also face structural barriers to supporting social connectedness among older adults, including transportation barriers, greater geographic distances, and access to technological connectivity. This research aims to discuss rural-specific risks of loneliness and social isolation among older adults, as well as rural/urban differences in loneliness and social isolation among older adults using the national COVID-19 Coping Study. Cross-sectional bivariate analyses highlight rural/urban differences in social activities during the pandemic. For example, rural older adults were more likely to use social media daily, compared with urban older adults (67% vs. 61%, p<0.05), but were less likely to have phone or video calls with others daily (21% vs. 26%, p<0.001). We will also share results of differences within rural older adults in loneliness, isolation, and social activities by socio-demographic characteristics in order to design targeted interventions to improve connectedness.

